# Risk of suicide and repeat self-harm after hospital attendance for non-fatal self-harm in Sri Lanka: a cohort study

**DOI:** 10.1016/S2215-0366(19)30214-7

**Published:** 2019-08

**Authors:** Duleeka Knipe, Chris Metcalfe, Keith Hawton, Melissa Pearson, Andrew Dawson, Shaluka Jayamanne, Flemming Konradsen, Michael Eddleston, David Gunnell

**Affiliations:** a Population Health Sciences, Bristol Medical School, University of Bristol, Bristol, UK; b South Asian Clinical Toxicology Research Collaboration (SACTRC), Faculty of Medicine, University of Peradeniya, Peradeniya, Sri Lanka; c Centre for Suicide Research, Department of Psychiatry, University of Oxford, Oxford, UK; d Pharmacology, Toxicology, and Therapeutics, University/BHF Centre for Cardiovascular Science, University of Edinburgh, Edinburgh, UK; e Central Clinical School, University of Sydney, Sydney, NSW, Australia; f Faculty of Medicine, University of Kelanyia, Kelanyia, Sri Lanka; g Department of Public Health, Faculty of Health and Medical Sciences, University of Copenhagen, Copenhagen, Denmark; h NIHR Biomedical Research Centre at the University Hospitals Bristol NHS Foundation Trust and the University of Bristol, Bristol, UK

## Abstract

**Background:**

Evidence from high income countries (HICs) suggests that individuals who present to hospital after self-harm are an important target for suicide prevention, but evidence from low and middle-income countries (LMICs) is lacking. We aimed to investigate the risk of repeat self-harm and suicide, and factors associated with these outcomes, in a large cohort of patients presenting to hospital with self-harm in rural Sri Lanka.

**Methods:**

In this cohort study, hospital presentations for self-harm at 13 hospitals in a rural area of North Central Province (population 224 000), Sri Lanka, were followed up with a self-harm surveillance system, established as part of a community randomised trial, and based on data from all hospitals, coroners, and police stations in the study area. We estimated the risk of repeat non-fatal and fatal self-harm and risk factors for repetition with Kaplan-Meier methods and Cox proportional hazard models. Sociodemographic (age, sex, and socioeconomic position) and clinical (past self-harm and method of self-harm) characteristics investigated were drawn from a household survey in the study area and data recorded at the time of index hospital presentation. We included all individuals who had complete data for all variables in the study in our primary analysis.

**Outcomes:**

Between July 29, 2011, and May 12, 2016, we detected 3073 episodes of self-harm (fatal and non-fatal) in our surveillance system, of which 2532 (82·3%) were linked back to an individual in the baseline survey. After exclusion of 145 ineligible episodes, we analysed 2259 index episodes of self-harm. By use of survival models, the estimated risk of repeat self-harm (12 months: 3· 1%, 95% CI 2·4–3·9; 24 months: 5·2%, 4·3–6·4) and suicide (12 months: 0·6%, 0·4–1·1; 24 months: 0·8%, 0·5–1·3) in our study was considerably lower than that in HICs. A higher risk of repeat self-harm was observed in men than in women (fatal and non-fatal; hazard ratio 2·0, 95% CI 1·3–3·2; p=0·0021), in individuals aged 56 years and older compared with those aged 10–25 years (fatal; 16·1, 4·3–59·9; p=0·0027), and those who used methods other than poisoning in their index presentation (fatal and non-fatal; 3·9, 2·0–7·6; p=0·00027). We found no evidence of increased risk of repeat self-harm or suicide in those with a history of self-harm before the index episode.

**Interpretation:**

Although people who self-harm are an important high-risk group, focusing suicide prevention efforts on those who self-harm might be somewhat less important in LMICs compared with HICs given the low risk of repeat self-harm and subsequent suicide death. Strategies that focus on other risk factors for suicide might be more effective in reducing suicide deaths in LMICs in south Asia. A better understanding of the low incidence of repeat self-harm is also needed, as this could contribute to prevention strategies in nations with a higher incidence of repetition and subsequent suicide death.

**Funding:**

Wellcome Trust.

## Introduction

Suicide is a major cause of mortality worldwide, with nearly 40% of deaths occurring in low-income and middle-income countries (LMICs) in the WHO south-east Asian region, despite this region containing only 26% of the global population.^1^ The epidemiology of suicide in LMICs differs from high income countries (HICs), with higher rates (deaths per 100 000 people per year) in young people (especially young women) and a narrower overall male to female rate ratio (1·6 in LMICs *vs* 3·5 in HICs).^1^

Evidence primarily from HICs suggests that previous non-fatal self-harm is the strongest risk factor for future suicide. An estimated 1·6% of those who self-harm will die by suicide in the subsequent year, and up to 3·9% in the subsequent 5 years.^2^ In the 1990s in the UK, 15% (95% CI 11–21) of people who died by suicide had attended an accident and emergency department for self-harm in the year before their death.^3^ Therefore, people who present to hospital after self-harm are an important target for suicide prevention efforts in HICs.^4^, ^5^ There is, however, a paucity of evidence from LMICs in south Asia regarding the risk of suicide after a self-harm episode.^2^ The few south Asian studies on this issue indicate that the risk of repeat self-harm is lower than in HICs,^6^, ^7^, ^8^, ^9^, ^10^ but there have been few prospective studies and most studies to date have been small and of low quality. A large prospective study^10^ restricted to people who had self-poisoned in a rural region of Sri Lanka reported that the risk of repeat self-poisoning at 12 months was 5·7% (95% CI 5·0–6·4) and of suicide (all methods) at 2 years was 0·7% (0·4–0·9), considerably lower than the risks reported in HICs.

**Research in context**

**Evidence before this study**

Hospital presenting self-harm is a high priority area for suicide prevention in high-income countries (HICs). A 2014 systematic review and meta-analysis suggested that 1·6% of individuals presenting to hospital with a self-harm episode go on to die by suicide in the next 12 months and 16% repeat self-harm. Only 4% of all identified studies in the review were from low-income and middle-income countries (LMICs), where 75% of all suicide deaths occur (with only one study from south Asia), and the review found that risk of repeat self-harm and subsequent suicide was lower in Asia than in other regions. Because of a paucity of good quality self-harm surveillance systems in LMICs, this association has been difficult to investigate. We updated the 2014 systematic review by searching PubMed for relevant studies published between Jan 1, 2012, and Feb 28, 2019, with the search terms (suicid*) OR (self AND harm) OR (self poisoning) AND MesH terms for “cohort studies” OR “follow up studies” OR hospital re-admission OR “longitudinal studies”, with no language restrictions. A 2019 large study investigating repeat self-poisoning in Sri Lanka reported a 12-month repeat rate of 5·7%, but focused on self-poisoning alone and did not investigate risk in relation to previous self-harm and the possible contribution of socioeconomic risk factors.

**Added value of this study**

By use of a self-harm surveillance register covering a population of more than 220 000 people in a south Asian LMIC (Sri Lanka), we estimated the risk of repeat self-harm and the risk of suicide to be considerably lower than in HICs. The risk of repeat self-harm and suicide was higher in men than in women but was not increased among those with a history of self-harm. To our knowledge, our study is the first sufficiently powered to study the risk of repeat self-harm and suicide after presentation with self-harm (all methods) in an LMIC. Only 8·5% of people who died by suicide had presented to hospital following self-harm in the previous 12 months. For the first time, to our knowledge, our study estimates the potential contribution of hospital-presenting self-harm and subsequent self-harm care to the overall burden of suicide in an LMIC.

**Implications of all the available evidence**

Although management and support of individuals who present to hospital with self-harm is a possible target for suicide prevention, the findings of this study suggest that it might be less important in a LMIC setting. Other strategies targeting more common risk factors (eg, access to lethal means, alcohol misuse, domestic violence, and poverty) might be more effective in reducing suicide mortality.

The aim of this study was to determine whether the aftercare of people presenting to hospital with self-harm should be an important priority for suicide prevention in LMICs. By use of data from a comprehensive suicide and self-harm surveillance system established as part of a large community cluster randomised controlled trial in rural Sri Lanka,^11^ we aimed to answer the following questions: what is the risk of repeat self-harm in individuals who present to hospital; what factors are associated with an increased risk of self-harm repetition; and are individuals who self-harm (non-fatally) at an increased risk of subsequent suicide?

## Methods

### Study design and participants

This cohort study was based in Sri Lanka, which has a population of 21 million. The randomised trial that formed the basis of this cohort study was done in the North Central Province of the country and recruited 95% of households (n=53 382; comprising 223 861 individuals) in the study area.^11^ The trial investigated the effectiveness of the provision of lockable pesticide storage containers in reducing the incidence of pesticide self-poisoning.^11^ Almost 80% of households in the study area engaged in some sort of farming activity.^11^ For this analysis, we included data that were collected in the self-harm surveillance system for the trial and could be linked to individuals included in the trial baseline survey. The baseline survey was done between Dec 31, 2010, and Feb 2, 2013, and included detailed face-to-face interviews in the local language in each house by trained interviewers, with one key informant per household and regular data quality checks.^11^, ^12^

We used the self-harm surveillance system to identify all episodes of non-fatal and fatal self-harm (regardless of intent) occurring in the study area and with individuals presenting to hospital^11^, ^12^ over the 3–5 year follow-up period (between July 29, 2011, and May 12, 2016) of the trial. Data were collected from 11 small peripheral hospitals and two larger referral hospitals where sicker patients were cared for. Self-harm cases were identified by a team of research assistants who visited medical, surgical, paediatric, and intensive care wards in the two large hospitals daily and the 11 peripheral hospitals at least on alternate days. We had details of the hospital the patient initially presented to for 2945 (95·8%) of the 3073 self-harm cases included in the study, 1993 (67·6%) of whom were initially admitted to one of the 11 peripheral hospitals; the number of cases per peripheral hospital varied between 12 and 358. Deaths from self-harm that occurred before presentation at hospital were identified through systematic surveys of police stations and coroners' records for the district. We use the term self-harm in this study to refer to any act of self-poisoning or self-injury, irrespective of motivation or suicidal intent. The study region was divided into ten areas (referred to as bands in the original),^11^ and random allocation and the pesticide storage box installation (the intervention evaluated in the trial from which data for this study were drawn) were rolled out in one area at a time, hence the variation in follow-up for each area. The surveillance system was developed in parallel to initiation of the trial in each area to ensure all self-harm episodes from study participants were identified after randomisation.

We included only episodes of self-harm that were linked back to an individual in the baseline survey and did not result in death at first episode in our analysis. Our cohort was restricted to individuals who were older than 10 years at the time of their first presentation to hospital with self-harm recorded on the surveillance system. As a quality assurance exercise, we revisited households 3 years after the start of the follow-up period and asked members to report on any self-harm attempts (fatal or non-fatal) that occurred in the household (appendix).

Ethics approval was received from the research ethics committees of the University of Peradeniya (Peradeniya, Sri Lanka) and Rajarata University of Sri Lanka (Mihintale, Sri Lanka). The chief village official was approached to seek consent for community enrolment; individual household verbal consent was then sought at the start of each household survey.

### Procedures

We investigated the risk of fatal and non-fatal repeat self-harm in relation to the following: demographic characteristics (sex and age at first presentation, with four age bands [10–25 years; 26–40 years; 41–55 years; and ≥56 years]); medical history or clinical data (data on previous self-harm before the baseline survey obtained via questioning the household respondent; method of self-harm collected from hospital records or by interviewing the patient or accompanying person); and socioeconomic characteristics collected in the baseline survey. Household socioeconomic position was measured on the basis of a household asset score.^13^ This score is a composite three-level measure derived by combining data on household construction and motorised vehicle ownership as follows: low (no motorised vehicle and poor quality household construction); middle (either a motorised vehicle or moderate or high quality household construction); or high (motorised vehicle ownership and moderate or high quality household construction). These variables are well-established risk factors for repeat self-harm and suicide in HICs and were well recorded in the trial forming the basis of this research,^11^, ^12^ as they were either primary outcomes or considered potential effect modifiers.

### Statistical analysis

We included all individuals who had complete data for all variables in the study in our primary analysis. We estimated the risks of all and fatal repeat self-harm at 12 months and 24 months with the Kaplan-Meier method. We used Cox proportional hazard models, with robust standard errors to accommodate any clustering of outcome events within villages, to determine the associations between patient characteristics (eg, age, sex, socioeconomic position, previous self-harm, and method of self-harm) and the risk of fatal and non-fatal repeat self-harm. In the original trial,^11^ there were no clear differences in the risk of self-harm between the intervention and control groups (relative risk 0·93, 95% CI 0·80–1·08; p=0·33), but we have taken the conservative approach of adjusting for the intervention arm in this analysis. The date of the index presentation was taken as time zero for the Cox proportional hazards models, with the time until first repeat episode of non-fatal self-harm, until fatal self-harm, or until censoring (end of study follow-up) recorded for each participant. The proportional hazards assumption was tested for each model using the estat phtest routine in Stata, which uses Schoenfeld residuals.^14^

In an exploratory analysis, we fitted a Weibull survival model without covariates to the time to repeat events, with the shape parameter taken as an indication of whether the risk of a further self-harm event was highest in the period immediately following the index event.

As an additional secondary analysis, we estimated the rate of hospital-presenting self-harm in the previous 12 months in those who died by suicide, compared with the rate of hospital-presenting self-harm in the previous 12 months in those who had not died by suicide at the end of the surveillance period (May 12, 2016). We used the full trial cohort dataset (223 861 participants), excluding those who were younger than 10 years at the time of the baseline survey, individuals with missing data, and individuals who had not been followed up for at least 12 months. We fitted age, sex, and intervention group adjusted logistic models with robust standard errors to account for the clustering of individuals within households.

We calculated the repetition risk excluding individuals who were reported as spending at least one month away from home in the year before the baseline survey. Given the size of the survey and scarce resources we did not collect detailed information regarding the reasons why people lived away from home. We added time away from home as a covariate and repeated the proportional hazard models to extend our sensitivity analysis.

All analyses were done with Stata version 15.1.

### Role of the funding source

The funder of the study had no role in study design, data collection, data analysis, data interpretation, or writing of the report. The corresponding author had full access to all the data in the study and had final responsibility for the decision to submit for publication.

## Results

**Figure 1:**
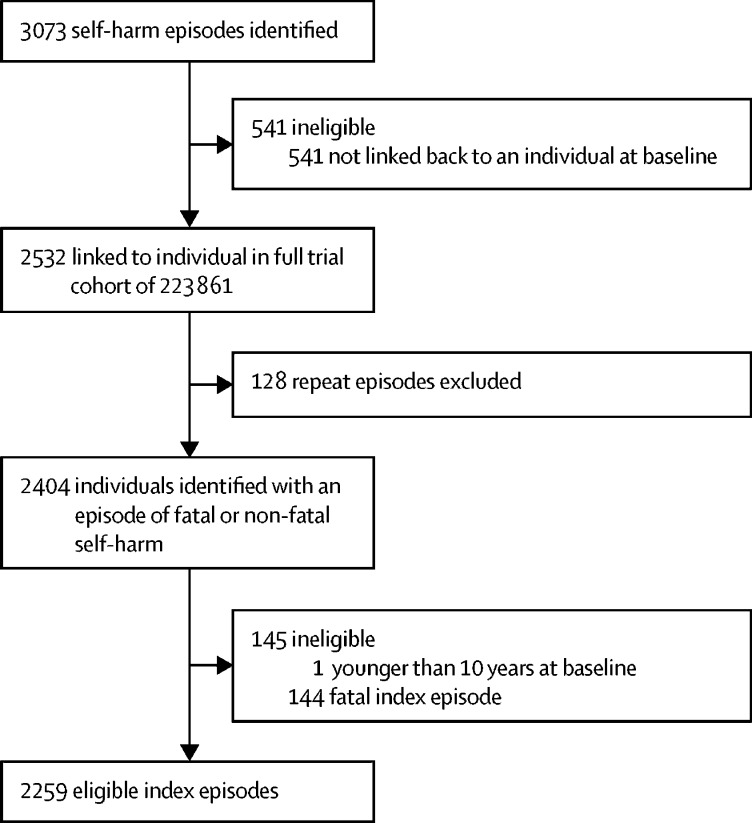
Cohort identification

Between July 29, 2011, and May 12, 2016, we detected 3073 episodes of self-harm (fatal and non-fatal) in our surveillance system, of which 2532 (82·3%) were linked back to an individual in the baseline survey (figure 1). These episodes occurred in 2404 individuals. We excluded one individual who was younger than 10 years at the time of index presentation. 144 of the 2403 index episodes resulted in death (figure 1), an overall case fatality of 6·0% (95% CI 5·1–7·0). 82 (6·4%) of 1272 pesticide poisoning episodes resulted in death, as did 14 (1·3%) of 1038 other poisoning episodes and 48 (51·6%) of 93 other methods of self-harm (hanging accounted for 35 [72·9%] of 48 other self-harm deaths). Therefore, we analysed 2259 eligible index episodes of self-harm. Self-poisoning was the most common method of non-fatal self-harm at first episode (2214 [98·0%] of 2259 patients), with more pesticide than non-pesticide self-poisoning episodes (table 1).

**Table 1:** HRs (95% CIs) based on robust standard errors for time to first self-harm repetition and time to death by suicide from index self-harm episode

		**n**	**First repeat self-harm, n (per 100 person-years)**	**Time to repeat self-harm**		**Fatal self-harm, n (per 100 person-years)**	**Time to death by suicide**	
				HR* (95% CI)	p value		HR* (95% CI)	p value
Overall		2259	116 (2·7)	..	..	16 (0·4)	..	..
Sex								
	Female	1136	38 (1·8)	1 (ref)	0·0021	1 (0)	1 (ref)	0·0093
	Male	1123	78 (3·6)	2·0 (1·3–3·2)	..	15 (0·7)	14·7 (1·9–111·9)	..
Age group, years								
	10–25	1238	72 (3·0)	1 (ref)	0·12†	4 (0·2)	1 (ref)	0·0027†
	26–40	655	34 (2·7)	0·9 (0·6–1·5)	..	7 (0·5)	3·3 (1·0–11·6)	..
	41–55	264	4 (0·8)	0·3 (0·1–0·8)	..	0 (0)	..	..
	≥56	102	6 (3·2)	1·1 (0·5–2·4)	..	5 (2·7)	16·1 (4·3–59·9)	..
Household socioeconomic position‡								
	High	1218	64 (2·8)	1 (ref)	0·68§	10 (0·4)	1 (ref)	0·18§
	Medium	842	45 (2·7)	1·0 (0·7–1·4)	..	3 (0·2)	0·4 (0·1–1·6)	..
	Low	199	7 (1·9)	0·7 (0·3–1·6)	..	3 (0·8)	1·9 (0·5–7·2)	..
Previous self-harm								
	No	2077	106 (2·7)	1 (ref)	0·93	15 (0·4)	1 (ref)	0·76
	Yes	182	10 (2·8)	1·0 (0·5–2·1)	..	1 (0·3)	0·7 (0·1–5·6)	..
Method of self-harm								
	Pesticide poisoning	1190	56 (2·4)	1 (ref)	0·00027§	13 (0·5)	1 (ref)	0·047§
	Other poisoning	1024	51 (2·7)	1·1 (0·7–1·7)	..	3 (0·2)	0·3 (0·1–1·0)	..
	Other method	45	9 (9·6)	3·9 (2·0–7·6)	..	0 (0)	..	..

P value refers to the risk of repetition (fatal or non-fatal) varied across the groups. HR=hazard ratio.

* Models adjusted for the intervention arm of the trial.^11^

† Testing for no trend.

‡ Household socioeconomic position measured by asset score.

§ Wald test comparing the model with and without the independent variable and using robust standard errors.

There were almost equal numbers of men and women in the study (table 1). 1238 (54·8%) of 2259 participants were aged 10–25 years at the time of index presentation. Previous self-harm (reported at the time of the baseline community survey) was recorded for 182 (8·1%) of 2259 individuals.

Our primary analysis was applied to 2259 individuals with index hospital presentation for non-fatal self-harm, and this cohort was followed up for a median of 1·9 years (range 0·003–4·8; IQR 0·9–2·8). 127 (111 non-fatal and 16 fatal) repeat self-harm episodes were reported in 116 individuals (maximum number of repeats was four). The rate of first repetition of self-harm over 4319 person-years of follow up was 2·7 per 100 person-years (95% CI 2·2–3·2). 61 individuals self-harmed again within 12 months of the index episode. By use of survival models, the estimated risk for repeat self-harm within 12 months was 3·1% (95% CI 2·4–3·9; table 2). 92 individuals self-harmed again within 24 months of the index episode, with a risk of repeat self-harm of 5·2% (95% CI 4·3–6·4; table 2). 13 deaths by suicide occurred within one year of the index episode of self-harm, and a further two deaths occurred within 2 years. We estimated the shape parameter for the Weibull survival model fitted to the time to repeat episodes to be 0·79 (95% CI 0·67–0·93), showing that the risk of a repeat episode is highest in the period immediately following the index event, with the risk diminishing over time.

**Table 2:** Kaplan-Meier estimates of 12-month and 24-month risk of repeat self-harm and death by suicide after index hospital presentation with self-harm

		n	**Self-harm repetition risk**		**Suicide death risk**	
			12 months	24 months	12 months	24 months
Overall		2259	3·1% (2·4–3·9), 61	5·2% (4·3–6·4), 92	0·6% (0·4–1·1), 13	0·8% (0·5–1·3), 15
Sex						
	Female	1136	2·1% (1·3–3·2), 20	3·1% (2·2–4·5), 28	0·1% (0·0–0·8), 1	0·1% (0·0–0·8), 1
	Male	1123	4·1% (3·0–5·5), 41	7·3% (5·7–9·2), 64	1·1% (0·7–2·1), 12	1·5% (0·9–2·5), 14
Age group, years						
	10–25	1238	3·3% (2·4–4·5), 36	5·2% (4·0–6·8), 52	0·3% (0·1–0·9), 4	0·3% (0·1–0·9), 4
	26–40	655	3·5% (2·3–5·4), 20	6·2% (4·4–8·8), 31	0·9% (0·4–2·2), 5	1·1% (0·5–2·5), 6
	41–55	264	0·4% (0·1–2·7), 1	1·7% (0·5–5·3), 3	..	..
	≥56	102	4·2% (1·6–10·8), 4	7·1% (2·9–16·9), 6	4·2% (1·6–10·8), 4	6·1% (2·5–14·6), 5
Household socioeconomic position*						
	High	1218	3·1% (2·3–4·4), 34	5·6% (4·3–7·4), 53	0·7% (0·4–1·5), 8	0·9% (0·4–1·7), 9
	Medium	842	3·1% (2·1–4·7), 23	4·8% (3·4–6·7), 32	0·4% (0·1–1·4), 3	0·4% (0·1–1·4), 3
	Low	199	2·4% (0·9–6·2), 4	4·7% (2·2–9·6), 7	1·0% (0·3–4·0), 2	1·7% (0·6–5·4), 3
Previous self-harm						
	No	2077	3·2% (2·5–4·1), 59	5·4% (4·4–6·6), 87	0·7% (0·4–1·2), 13	0·8% (0·5–1·4), 14
	Yes	182	1·3% (0·3–5·1), 2	3·6% (1·5–8·4), 5	..	0·7% (0·1–5·0), 1
Method of self-harm						
	Pesticide poisoning	1190	2·8% (1·9–3·9), 30	4·8% (3·6–6·4), 45	0·9% (0·5–1·7), 10	1·2% (0·7–2·1), 12
	Other poisoning	1024	3·1% (2·1–4·5), 27	5·1% (3·8–7·0), 40	0·4% (0·1–1·2), 3	0·4% (0·1–1·2), 3
	Other method	45	9·6% (3·7–23·6), 4	17·8% (8·9–33·9), 7	..	..

Data are risk (95% CI), n, unless otherwise indicated.

* Household socioeconomic position measured by asset score.

**Figure 2:**
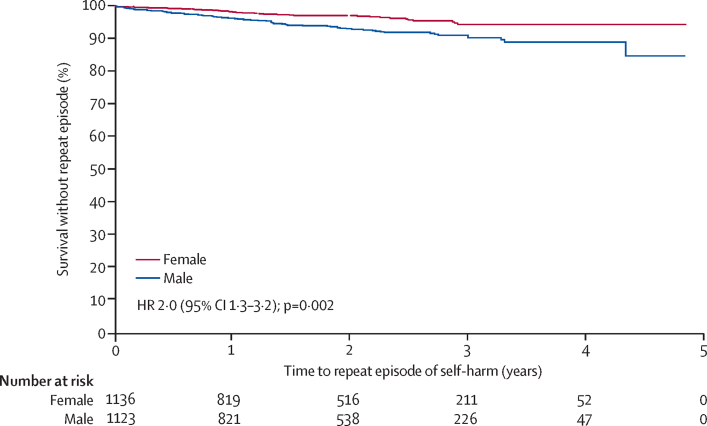
Kaplan-Meier graph of time to the first non-fatal repeat episode of self-harm by sex

The risk of repeat self-harm was higher in men than in women (hazard ratio [HR] 2·0, 95% CI 1·3–3·2; p=0·0021) and in those who used other methods of self-harm for their first episode compared with those who used pesticides (4·0, 2·0–7·6; p=0·00027; figure 2; table 1). We found evidence against the proportional hazards assumption for the model investigating the association between previous history of self-harm and non-fatal repetition (p=0·020), but inspection of the Kaplan-Meier curves gave no suggestion of the estimated hazard ratio of 1·0 being misleading (data not shown). Self-cutting (23 [51%] of 45 participants) and attempted hanging (15 [33%] of 45 participants) were the most common other methods used in a first episode of self-harm.

People generally used the same method in their repeat self-harm episode as in their index self-harm episode. 56 individuals who repeated self-harm self-poisoned with pesticides at the index episode, and 35 (62·5%) of these used this same method in the second episode. 51 individuals who repeated self-harm self-poisoned with a non-pesticide substance at the index episode, and 32 (62·7%) of these used the same method at the second episode. Nine individuals used a method other than self-poisoning in the index episode, and four (44·4%) of these used a non-self-poisoning method in the second episode.

There were 16 deaths by suicide after index hospital presentation for non-fatal self-harm. The risk of fatal self-harm after index presentation with self-harm was 0·6% (95% CI 0·4–1·1) within 12 months and 0·8% (0·5–1·3) within 24 months (table 2). The risk of suicide was most increased in men (HR 14·7, 95% CI 1·9–111·9; p=0·0093) and in those aged 56 years and older (16·1, 4·3–59·9; p=0·0027) at the time of their index episode (table 1).

We did a sensitivity analysis to explore whether the risk of repeat self-harm and suicide was similar in 1553 (69%) of 2259 study cohort members who reported spending less than 1 month away from their home, compared with the findings for the entire study cohort. Reasons for spending time away from the home included working on agricultural land, living at another address, or working at some distance from the home (eg, garment factory worker). The risk of non-fatal repeat self-harm and suicide (in those residing in their homes at least 11 months of the previous year) at 12 months (self-harm repetition 3·1%; suicide death 0·7%) and 24 months (self-harm repetition 5·3%; suicide death 0·9%) was consistent with our main analysis findings. Results from the proportional hazard models when the proportion of time away from home was included as a covariate did not alter our conclusions (data not shown).

160 deaths by suicide were recorded on the self-harm surveillance system over the study period, only 16 (10·0%) of which occurred after an episode of self-harm recorded on the surveillance register. 118 (73·8%) of 160 deaths by suicide occurred after the first 12 months of surveillance follow-up. In a secondary analysis, we compared these individuals who died by suicide with individuals older than 10 years at the time of the baseline survey who had not died by suicide at the end of the surveillance period and had complete data for the variables of interest. In the 12 months before death by suicide, 10 (8·5%) of 118 individuals who died by suicide (95% CI 4·1–15·0) presented to hospital with self-harm, compared with 598 (0·3%) of 183 277 individuals (3·3 per 1000 individuals; 95% CI 3·0–3·5) who presented to hospital with self-harm but did not die by suicide in the 12 months before the end of surveillance. People who died by suicide were 36·7 times (95% CI 18·7–71·7) more likely to have presented to hospital with self-harm in the 12 months before their death compared with those who did not die by suicide (adjusted for age, sex, and intervention arm; appendix).

To check for episodes of self-harm that did not result in presentation to hospital and assess levels of emigration from the study area, the household survey team revisited 13 999 (26·2%) of 53 382 households in the study area between Jan 31, 2015, and July 31, 2016, and found only 269 self-harm attempts were reported to have occurred between the time of the baseline survey and resurvey by household members, of which 247 (91·8%) were reported to have presented to hospital. Emigration from the study area during the trial follow-up period was modest (6520 [11%] of 59 025 residents of revisited households).

## Discussion

To our knowledge, this is the largest prospective study from a LMIC in south Asia to estimate the risk of repeat self-harm by any method. We observed a 3·1% risk (95% CI 2·4–3·9) of repeat self-harm and a 0·6% risk (0·4–1·1) of death by suicide at 12 months after index hospital presentation with self-harm. We observed a 2 times higher risk of non-fatal and fatal repeat self-harm in men compared with women and a 16 times higher risk of fatal self-harm in people aged 56 years or older versus those aged 10–25 years at index case hospital presentation. We found evidence that the risk of self-harm repetition was higher in those who used methods other than self-poisoning (HR 3·9) at their index presentation, compared with those who self-poisoned with pesticides. We found no evidence that previous self-harm increased the risk of repeat self-harm or suicide. We found that 8·5% of individuals who died by suicide in this study had presented to hospital with self-harm in the 12 months before their death. People who died by suicide were 36·7 times more likely to have presented to hospital with self-harm 12 months before their death compared with those who did not die by suicide.

The risk of repeat non-fatal self-harm in this study was considerably lower than that reported in a 2014 systematic review and meta-analysis of repetition of hospital presenting self-harm,^2^ with no overlap in the 95% CIs for the corresponding estimates from the two studies (table 3).^2^ This systematic review^2^ identified only seven papers (4% of all identified research) from LMICs, with only one study from south Asia.^7^ We observed a higher risk of repeat self-harm in men compared with women (HR 2·0, 95% CI 1·3–3·2). The systematic review^2^ found no difference between men and women, but a more recent large follow-up study^10^ of people presenting to hospital after self-poisoning in Sri Lanka also reported an increased risk of repeat self-poisoning in men compared with women (odds ratio 1·3, 95% CI 1·0–1·6). The risk of death by suicide 12 months after the index episode was also substantially lower in our study compared with the systematic review estimate^2^ (table 3), but was more similar to the 2-year suicide risk of 0·7% (95% CI 0·4–0·9) reported by Pushpakumara and colleagues.^10^ Our findings of an elevated risk of suicide in men compared with women after index self-harm presentation are consistent with the systematic review.^2^

**Table 3:** Estimated risk of repeat self-harm (non-fatal and fatal) in the present study compared with the Carroll et al meta-analysis^2^

	**12-month risk (95% CI)**	**24-month risk (95% CI)**
**Non-fatal self-harm**		
This study	3·1% (2·4–3·9)	5·2% (4·3–6·4)
Carroll et al, 2014^2^	16·3% (15·1–17·7)	16·8% (14·7–19·2)
**Fatal self-harm**		
This study	0·6% (0·4–1·1)	0·8% (0·5–1·3)
Carroll et al, 2014^2^	1·6% (1·2–2·4)	2·1% (1·6–2·8)

A possible reason for the lower observed risk of repeat self-harm in Asia is that pesticide self-poisoning has a high case fatality rate, thereby removing those at a higher risk of repetition.^6^, ^15^, ^16^ Over the past three decades there have been a series of pesticide bans, most recently in 2008–11, which have contributed to a reduction in the number of pesticide-related deaths by suicide in Sri Lanka.^17^, ^18^, ^19^ These bans have resulted in marked reductions in the case fatality associated with pesticide self-poisoning. In this study, we observed a case fatality rate of 6%, substantially lower than that previously observed in national and secondary hospital data (11–12%),^20^, ^21^ and supporting the positive effect of pesticide regulations on suicide mortality trends. Even if the 144 (6·4%) individuals who died on their index presentation survived and went on to repeat self-harm within 12 months, the 12-month repetition rate would be only 8·5%, which is substantially lower than that in HICs. Other possible reasons for the low prevalence of repeat self-harm in Asia are the lower prevalence of mental disorders among those who self-harm compared to levels seen in HICs^22^ and longer inpatient stays helping individuals get through the period of greatest risk of repeat self-harm. Our estimates of a lower rate of self-harm repetition are consistent with previous estimates of repetition from south Asia,^6^, ^8^, ^9^, ^10^ but lower than the 12-month repetition rate from a prospective study from India (14%, 95% CI 10–19).^7^ Possible explanations for these differences might be differing methodology in the Indian study, including collection of data on self-reported self-harm, or differences in the socioeconomic and psychological health of our study population.

Previous studies have shown that the risk of repeat self-harm is higher in individuals who present with self-cutting compared with self-poisoning.^23^, ^24^ This finding is consistent with our study results, as we observed a greatly elevated risk of repeat self-harm after index presentations with other methods of self-harm (which included a large proportion of self-cutting).

Compared with previous research, we observed a lower risk of self-harm in the year before death by suicide (8·5% *vs* 15%^3^). However, when compared with those who did not die by suicide in this study, a previous self-harm attempt (in the last 12 months) was associated with an increased risk of suicide. Although those who present to hospital with self-harm are an attractive target for suicide prevention (assuming a causal relationship) only 8·5% (population attributable fraction) of individuals who died by suicide had presented to hospital with self-harm in our study.

Our study has some limitations that should be considered when interpreting the findings. First, the study focused on non-fatal self-harm that resulted in presentation to hospital. Some self-harm might not result in hospital presentation, and this would particularly be the case for non-poisoning episodes like self-cutting; however, our follow up household survey findings suggested that our estimates of repeat self-harm are unlikely to be greatly underestimated. Second, the surveillance register collected restricted clinical and diagnostic data, which limits our ability to explore these as risk factors for repetition. Third, we do not know whether individuals presented to hospital for repeat self-harm outside the study area. However, the household resurvey suggested that emigration from the study area during the trial follow-up period was modest (11%) and every attempt was made to collect cases from hospitals that were close to the study area, minimising missing cases.^11^ Fourth, because the study was done in a rural area where 80% of households were involved in farming and 98% of self-harm episodes involved self-poisoning, most often by pesticides, the findings might be less applicable to urban LMIC settings, where the socioeconomic circumstances and methods used for self-harm are different, and to rural LMIC settings that use different farming methods. Finally, there were only 16 suicide deaths in those who presented to hospital with an index self-harm episode. Therefore, the study is probably underpowered to detect anything but a large difference in risk for the potential risk variables that we investigated for suicide.

We estimate that 5·2% of patients who have self-harmed will self-harm again within 2 years of an index hospital presentation in Sri Lanka. An estimated 8 (0·8%) of every 1000 individuals will go on to die by suicide in the same time period. Although the rate of previous self-harm (at 12 months) in those who died by suicide in this study was lower than in HICs, hospital presenting self-harm remains a substantial risk factor for suicide; nevertheless, the population attributable risk for this exposure was only 8·5%. Based on the findings of this study, focusing suicide prevention efforts on those who self-harm might be somewhat less important in LMICs compared with HICs given the low risk of repeat self-harm and death by suicide. Strategies that focus on other risk factors for suicide (eg, access to lethal means, domestic violence, harmful alcohol use), or on improved mental health support or welfare support, might be more effective in reducing suicide deaths in LMICs in south Asia. Further research investigating the reasons underlying the low rate of repeat self-harm is needed, as a better understanding of this phenomenon might contribute to prevention strategies in nations with a higher incidence of repeat self-harm and subsequent death by suicide.

## Data sharing

Data are available from study authors on request.
