# Supplementary material for: Risk of suicide and repeat self-harm after hospital attendance for non-fatal self-harm in Sri Lanka: a cohort study
Source: Lancet Psychiatry. 2019 Aug;6(8):659–66. doi: 10.1016/S2215-0366(19)30214-7 (PMC6639451; doi:10.1016/S2215-0366(19)30214-7)
Supplement: Supplementary appendix [file mmc1.pdf]

THE LANCET
Psychiatry

Supplementary appendix

This appendix formed part of the original submission and has been peer reviewed. We post it as supplied by the authors.

Supplement to: Knipe D, Metcalfe C, Hawton K, et al. Risk of suicide and repeat self-harm after hospital attendance for non-fatal self-harm in Sri Lanka: a cohort study. *Lancet Psychiatry* 2019; published online July 1. http://dx.doi.org/10.1016/S2215-0366(19)30214-7.

Supplementary Table. Parameter estimates for the logistic regression model comparing the odds of having presented to hospital following self-harm in the 12 month previous to a fatal self-harm event, or reaching the end of study follow-up alive

	Odds ratio	95% confidence interval*
Admission following self-harm in previous 12 months	36.62	18.69, 71.75
Male	4.37	2.77, 6.92
Age (per year older)	1.02	1.01, 1.03
Trial intervention arm	1.08	0.75, 1.55

***Based on robust standard errors**

Supplementary Information: Method of selection of households participating in follow-up survey

Recruitment of the 26% of households / areas participating in the follow-up survey was carried following the same sequence as for recruitment to the randomised trial; i.e. all households surveyed in the first area to be recruited to the trial were re-surveyed first, the repeat survey team then moved to the second area etc. After follow-up data collection had been completed in two areas (13,999 Households) the data monitoring committee reviewed any differential case ascertainment and found no evidence of any difference between the study arms. Thus, a decision to cease follow-up survey was made.
